# An Entropy-Based Approach for Measuring Factor Contributions in Factor Analysis Models

**DOI:** 10.3390/e20090634

**Published:** 2018-08-24

**Authors:** Nobuoki Eshima, Minoru Tabata, Claudio Giovanni Borroni

**Affiliations:** 1 Center for Educational Outreach and Admissions, Kyoto University, Kyoto 606-8501, Japan; 2 Department of Mathematical Sciences, Osaka Prefecture University, Osaka 599-8532, Japan; 3 Department of Statistics and Quantitative Methods, University of Milano Bicocca, 20126 Milano, Italy

**Keywords:** entropy coefficient of determination, factor contribution, factor loading, path analysis

## Abstract

In factor analysis, factor contributions of latent variables are assessed conventionally by the sums of the squared factor loadings related to the variables. First, the present paper considers issues in the conventional method. Second, an alternative entropy-based approach for measuring factor contributions is proposed. The method measures the contribution of the common factor vector to the manifest variable vector and decomposes it into contributions of factors. A numerical example is also provided to demonstrate the present approach.

## 1. Introduction

Factor analysis is a statistical method for extracting simple structures to explain inter-relations between manifest and latent variables. The origin dates back to the works of [1], and the single factor model was extended to the multiple factor model [2]. These days, factor analysis is widely applied in behavioral sciences [3]; hence, it is important to interpret the extracted factors and is critical to explain how such factors influence manifest variables, that is, measurement of factor contribution. Let $X_{i}$ be manifest variables; $\xi_{j}$ latent variables (common factors); $\varepsilon_{i}$ unique factors related to $X_{i}$; and let $\lambda_{ij}$ be factor loadings that are weights of factors $\xi_{j}$ to explain $X_{i}$. Then, the factor analysis model is given as follows:

(1) $$\;X_{i}=\sum_{j=1\;}^{m}\lambda_{ij}\xi_{j}+\varepsilon_{i},\;\;i=1,2,\ldots ,p,\;$$

where

$$E\left(X_{i}\right)=E\left(\xi_{j}\right)=E\left(\varepsilon_{i}\right)=0,\;\mathrm{var}\left(\xi_{j}\right)=1,\;\mathrm{cov}\left(\xi_{j},\varepsilon_{i}\right)=0,\;\mathrm{cov}\left(\varepsilon_{i},\varepsilon_{k}\right)=0\;\mathrm{for}\;i\neq k\;\mathrm{and}\;\mathrm{var}\left(\varepsilon_{i}\;\right)=\sigma_{i}^{2}>0$$

For the simplicity of discussion, common factors $\xi_{j}\;$are assumed to be mutually independent in this section, that is, we first consider an orthogonal factor analysis model. In the conventional approach, the contribution of factor $\xi_{j}$ to all manifest variables$\;X_{i}$, $C_{j}$, is defined as follows:

(2) $$\;C_{j}=\sum_{i=1\;}^{p}\mathrm{cov}{\left(X_{i},\xi_{j}\right)}^{2}=\sum_{i=1}^{p}\lambda_{ij}^{2}$$

The above definition of factor contributions is based on the following decomposition of the total of variances of the observed variables$\;X_{i}$ [4] (p. 59):

$$\;\sum_{i=1\;}^{p}\mathrm{var}\left(X_{i}\right)=\sum_{j=1}^{m}\sum_{i=1}^{p}\lambda_{ij}^{2}+\sum_{i=1}^{p}\sigma_{i}^{2}$$

What physical meaning does the above quantity have? Applying it to the manifest variables observed, however, such a decomposition leads to scale-variant results. For this reason, factor contribution is usually considered on the standardized versions of manifest variables$\;X_{i}$. What does it mean to measure factor contributions by (2)? For standardized manifest variables$\;X_{i}$, we have

(3) $$\;\lambda_{ij\;}=\mathrm{cor}\left(X_{i},\xi_{j}\right)$$

Then, (2) is the sum of the coefficients of determination for all standardized manifest variables$\;X_{i}\;$with respect to a single latent variable$\;\xi_{j}$. The squared correlation coefficients (3), that is, $\mathrm{cor}{\left(X_{i},\xi_{j}\right)}^{2}$, are the ratios of explained variances of a manifest variable$\;X_{i}$, and in this sense, they can be interpreted as the contributions (effects) of factors $\;\xi_{j}$ to the manifest variable $X_{i}$. Although, what does the sum of these with respect to all manifest variables $X_{i}$, that is, (2), mean? The conventional method may be intuitively reasonable for measuring factor contributions; however, we think it is sensible to propose a method measuring factor contributions as the effects of factors on the manifest variable vector $X=\left(X_{1},X_{2},\ldots ,X_{p}\right)$, which are interpretable and have a theoretical basis. There is no research on this topic as far as we have searched. The present paper provides an entropy-based solution to the problem. Entropy is a useful concept to measure the uncertainty in the systems of random variables and sample spaces [5] and it can be applied to measure multivariate dependences of random variables [6,7].

This paper proposes an entropy-based method for measuring factor contributions of $\;\xi_{j}$ to the manifest variable vector $\;X=\left(X_{1},X_{2},\ldots ,X_{p}\right)$ concerned, which can treat not only orthogonal factors, but also oblique cases. The present paper has five sections in addition to this section. In Section 2, the conventional method for measuring factor contributions is reviewed. Section 3 considers the factor analysis model in view of entropy and makes a preliminary discussion on measurement of factor contribution. In Section 4, an entropy-based path analysis is applied as a tool to measure factor contributions. Contributions of factors $\xi_{j}\;$are defined by the total effects of the factors on the manifest variable vector, and the contributions are decomposed into those to manifest variables and subsets of manifest variables. Section 5 illustrates the present method using a numerical example. Finally, in Section 6, some conclusions are provided.

## 2. Relative Factor Contributions in the Conventional Method

In the conventional approach, for the orthogonal factor model (1), the contribution ratio of $\xi_{j}$ is defined by

(4) $$\;{\mathrm{RC}\;}_{j}=\frac{C_{j}}{\sum_{l=1}^{m}C_{l}}=\frac{\sum_{i=1}^{p}\lambda_{ij}^{2}.}{\sum_{l=1}^{m}\sum_{k=1}^{p}\lambda_{kl}^{2}}$$

The above measure is referred to as the factor contribution ratio in the common factor space. Let $R_{i}\;$ be the multiple correlation coefficient of latent variable vector $\xi ={\left(\xi_{1},\xi_{2},\ldots ,\xi_{m}\right)}^{T}$ and manifest variable$\;X_{i}$. Then, for standardized manifest variable$\;X_{i}$, we have

(5) $$\;R_{i}^{2}=\sum_{j=1\;}^{m}\lambda_{ij}^{2}$$

The above quantity can be interpreted as the effect (explanatory power) of latent variable vector $\xi =\left(\xi_{j}\right)\;$on manifest variable$\;X_{i}$; however, the denominator of (4) is the sum of those effects (5) and there is no theoretical basis to interpret it. Another contribution ratio of$\;\xi_{j}\;$is referred to as that in the whole space of $X=\left(X_{i}\right)$, and is defined by

(6) $$\;{\tilde{\mathrm{RC}\;}}_{j}=\frac{C_{j}}{\sum_{i=1}^{p}\mathrm{var}\left(X_{i}\right)}=\frac{\sum_{k=1}^{p}\lambda_{kj}^{2}}{\sum_{k=1}^{p}\left(\sum_{l=1}^{m}\lambda_{kl}^{2}+\sigma_{k}^{2}\right)}$$

If the manifest variables are standardized, we have

$$\;{\tilde{\mathrm{RC}\;}}_{j}=\frac{C_{j}}{p}=\frac{\sum_{k=1}^{p}\lambda_{kj}^{2}}{p}$$

Here, there is an issue similar to (4), because the denominator in (6) does not express the variation of the manifest variable vector $X=\left(X_{i}\right)$. Indeed, it is the sum of the variances of manifest variables and does not include covariances between them. In the next section, the factor analysis model (1) is reconsidered in the framework of generalized linear models (GLMs), and the effects (contributions) of latent variables $\xi_{j}$ on the manifest variable vector $X=\left(X_{i}\right)$, that is, factor contributions, are discussed through entropy [8].

## 3. Factor Analysis Model and Entropy

It is assumed that factors $\varepsilon_{i}$ and $\xi_{j}$ are normally distributed, and the factor analysis model (1) is reconsidered in the GLM framework. Let $\Lambda =\left(\lambda_{ij}\right)$ be a p×m factor loading matrix; let Φ be an m×m correlation matrix of common factor vector $\xi ={\left(\xi_{1},\xi_{2},\ldots ,\xi_{m}\right)}^{T}$; and let Ω be the p×p variance-covariance matrix of unique factor vector $\varepsilon ={\left(\varepsilon_{1},\varepsilon_{2},\ldots ,\varepsilon_{p}\right)}^{T}$. The conditional density function of X given ξ, f(x|ξ), is normal with mean Λξ and variance matrix Ω, and is given as follows:

$$\;f\left(x|\xi \;\right)=\frac{1}{{\left(2\pi \right)}^{\frac{p}{2}}{\left|\Omega \right|}^{\frac{1}{2}}}\exp\left(\frac{X^{T}\tilde{\Omega }\Lambda \xi -\frac{1}{2}\xi^{T}\Lambda^{T}{\tilde{\Omega }}^{2}\Lambda \xi }{\left|\Omega \right|}-\frac{\frac{1}{2}X^{T}\tilde{\Omega }X}{\left|\Omega \right|}\right)$$

where $\;\tilde{\Omega }$ is the cofactor matrix of  Ω. Let f(x) and g(ξ) be the marginal density functions of X and  ξ, respectively. Then, a basic predictive power measure for GLMs [9] is based on the Kullback–Leibler information [6], and applying it to the above model, we have

(7) $$\;\mathrm{KL}\left(X,\;\xi \;\right)=\iint f\left(x|\xi \right)g\left(\xi \right)\log\frac{f\left(x|\xi \right)}{f\left(x\right)}dxd\xi +\iint f\left(x\right)g\left(\xi \right)\log\frac{f\left(x\right)}{f\left(x|\xi \right)}dxd\xi =\frac{\mathrm{tr}\tilde{\Omega }\Lambda \Phi \Lambda^{T}}{\left|\Omega \right|}$$

The above measure was derived from a discussion on log odds ratios in GLMs [9], and is scale-invariant with respect to manifest variables $\;X_{i}$. The numerator of (7) is the explained entropy of X by  ξ, and the denominator is the dispersion of the unique factors in entropy, that is, the generalized variance of $\;\varepsilon ={\left(\varepsilon_{1},\varepsilon_{2},\ldots ,\varepsilon_{p}\right)}^{T}$. Thus, (7) expresses the total effect (contribution) of factor vector $\xi =\left(\xi_{j}\right)\;$on manifest variable vector $\;X=\left(X_{i}\right)\;$ in entropy, and is denoted by C(ξ→X) in the present paper. The entropy coefficient of determination (ECD) is calculated as follows [9]:

(8) $$\;\mathrm{ECD}\left(X,\;\xi \;\right)=\frac{\mathrm{tr}\tilde{\Omega }\Lambda \Phi \Lambda^{T}}{\mathrm{tr}\tilde{\Omega }\Lambda \Phi \Lambda^{T}+\left|\Omega \right|}$$

The denominator of the above measure is interpreted as the variation of manifest variable vector $\;X=\left(X_{i}\right)$ in entropy and the numerator is the explained variation of random vector X in entropy. In this sense, ECD (8) is the factor contribution ratio of $\xi =\left(\xi_{j}\right)$ for the whole entropy space of $X=\left(X_{i}\right)$, and it expresses the standardized total effect of $\xi ={\left(\xi_{1},\xi_{2},\ldots ,\xi_{m}\right)}^{T}$ on the manifest variable vector $\;X={\left(X_{1},X_{2},\ldots ,X_{p}\right)}^{T}$, which is denoted by $e_{T}\left(\xi \to X\right)$ [8,10]. As for (6), in the present paper, the ECD is denoted by $\tilde{\mathrm{RC}}\left(\xi \to X\right)$, that is, the relative contribution of factor vector ξ for the whole space of manifest variable vector  X in entropy.

**Remark** **1.** Let Σ be the p×p variance-covariance matrix of manifest variable vector $X={\left(X_{1},X_{2},\ldots ,X_{p}\right)}^{T}$ and let Φ be the m×m correlation matrix of ξ. Then, we have

(9) $$\;\Sigma =\;\Lambda \Phi \Lambda^{T}+\Omega \;$$

For assessing the goodness-of-fit of the models, the following overall coefficient of determination (OCD) is suggested ([11], p. 60) on the basis of (9):

$$\;\mathrm{OCD}\left(X,\;\xi \;\right)=1-\frac{\left|\Omega \right|}{\left|\Sigma \right|}\left(=\frac{\left|\Sigma \right|-\left|\Omega \right|}{\left|\Sigma \right|}\right)$$

Determinant  |Ω|  is the generalized variance of unique factor vector $\varepsilon ={\left(\varepsilon_{1},\varepsilon_{2},\ldots ,\varepsilon_{p}\right)}^{T}$ and |Σ| is that of manifest variable vector $X={\left(X_{1},X_{2},\ldots ,X_{p}\right)}^{T}$. Then, OCD is interpreted as the ratio of the explained generalized variance of manifest variable vector $X={\left(X_{1},X_{2},\ldots ,X_{p}\right)}^{T}$ by common factor vector $\xi ={\left(\xi_{1},\xi_{2},\ldots ,\xi_{m}\right)}^{T}\;$in the *p*-dimensional Euclidian space. On the other hand, from (8), it follows that

$$\;\mathrm{ECD}\left(X,\;\xi \;\right)=1-\frac{\left|\Omega \right|}{tr\tilde{\Omega }\Lambda \Phi \Lambda^{T}+\left|\Omega \right|}$$

Hence, ECD is viewed as the ratio of the explained variation of the manifest variable vector in entropy.

Cofactor matrix $\tilde{\Omega }\;$is diagonal and the (i,i) elements are $\underset{k\neq i}{\prod }\sigma_{k}^{2},\;i=1,2,\ldots ,p$. If common factors are statistically independent, it follows that

$$\;\mathrm{tr}\tilde{\Omega }\Lambda \Phi \Lambda^{T}=\sum_{i=1}^{p}\underset{k\neq i}{\prod }\sigma_{k}^{2}\sum_{j=1}^{m}\lambda_{ij}^{2}\;\;=\sum_{i=1\;}^{p}\sum_{j=1}^{m}\lambda_{ij}^{2}\underset{k\neq i}{\prod }\sigma_{k}^{2}$$

Thus, (7) is decomposed as

$$\;\mathrm{KL}\left(X,\;\xi \;\right)=\sum_{i=1}^{p}\sum_{j=1}^{m}\frac{\lambda_{ij}^{2}}{\sigma_{i}^{2}}$$

As detailed below, in the present paper, the contribution of factor $\xi_{j}$ to X, $C\left(\xi_{j}\to X\right)$, is defined by

(10) $$\;C\left(\xi_{j}\to X\;\right)=\sum_{i=1}^{p}\frac{\lambda_{ij}^{2}}{\sigma_{i}^{2}}$$

**Remark** **2.** The above contribution is different from the conventional definition of factor contribution (2); unless $\sigma_{i}^{2}=1,\;i=1,2,\ldots ,p$. In this sense, we may say that the standardization of manifest variables in entropy is obtained by setting all the unique factor variances to one.

In the next section, the contributions (effects) of factors$\;\xi_{j}\;$to manifest variable vector X are discussed in a general framework through an entropy-based path analysis [8].

## 4. Measurement of Factor Contribution Based on Entropy

**Figure 1:**
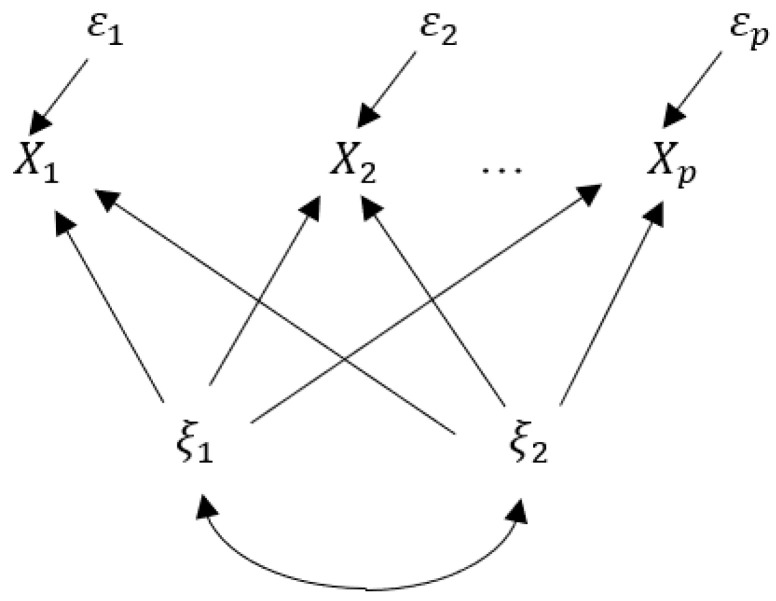
Path diagram for factor analysis model (1) (m=2).

A path diagram for the factor analysis model is given in Figure 1, in which the single-headed arrows imply the directions of effects of factors and the double-headed curved arrows indicate the associations between the related variables. In this section, common factors are assumed to be correlated, that is, we consider an oblique case, and an entropy-based path analysis [8] is applied to make a general discussion in the measurement of factor contributions.

**Theorem** **1.** *In the factor analysis model (1),*

$$\;\mathrm{KL}\left(X,\xi \;\right)=\sum_{i=1}^{p}\mathrm{KL}\left(X_{i},\xi \right)$$

**Proof.**  Let $f_{i}\left(x_{i}|\xi \right)$ be the conditional density functions of manifest variables $X_{i}$, given factor vector ξ; let $f_{i}\left(x_{i}\right)$ be the marginal density functions of $X_{i}$; let f(x) be the marginal density function of X; and let g(ξ) be the marginal density function of common factor vector ξ. As the manifest variables are conditionally independent, given factor vector ξ, the conditional density function of X is

$$f\left(x|\xi \;\right)=\prod_{i=1}^{p}f_{i}\left(x_{i}|\xi \right)$$

From (7), we have

$$\;\mathrm{KL}\left(X,\xi \;\right)=\;\iint \prod_{i=1}^{p}f_{i}\left(x_{i}|\xi \right)g\left(\xi \right)\log\frac{\prod_{k=1}^{p}f_{k}\left(x_{k}|\xi \right)}{f\left(x\right)}dxd\xi +\;\;\iint f\left(x\right)g\left(\xi \right)\log\frac{f\left(x\right)}{\prod_{k=1}^{p}f_{k}\left(x_{k}|\xi \right)}dxd\xi$$

$$\begin{array}{l} \;=\iint \left(\prod_{i=1\;}^{p}f_{i}\left(x_{i}|\xi \right)g\left(\xi \right)-f\left(x\right)g\left(\xi \right)\right)\log\prod_{k=1}^{p}f_{k}\left(x_{k}|\xi \right)dxd\xi \\ \;=\iint \prod_{i=1\;}^{p}f_{i}\left(x_{i}|\xi \right)g\left(\xi \right)\log\frac{\prod_{k=1}^{p}f_{k}\left(x_{k}|\xi \right)}{\prod_{k=1}^{p}f_{k}\left(x_{k}\right)}dxd\xi \\ \;+\iint f(x)\;g\left(\xi \right)\log\frac{\prod_{k=1}^{p}f_{k}\left(x_{k}\right)}{\prod_{k=1}^{p}f_{k}\left(x_{k}|\xi \right)}dxd\xi \end{array}$$

$$\;=\sum_{k=1\;}^{p}\iint \prod_{i=1}^{p}f_{i}\left(x_{i}|\xi \right)g\left(\xi \right)\log\frac{f_{k}\left(x_{k}|\xi \right)}{f_{k}\left(x_{k}\right)}dxd\xi +\sum_{k=1}^{p}\iint f\left(x\right)g\left(\xi \right)\log\frac{f_{k}\left(x_{k}\right)}{f_{k}\left(x_{k}|\xi \right)}dxd\xi \;\;=\sum_{k=1\;}^{p}\iint f_{k}\left(x_{k}|\xi \right)g\left(\xi \right)\log\frac{f_{k}\left(x_{k}|\xi \right)}{f_{k}\left(x_{k}\right)}dx_{k}d\xi \;\;+\sum_{k=1\;}^{p}\iint f_{k}\left(x_{k}\right)g\left(\xi \right)\log\frac{f_{k}\left(x_{k}\right)}{f_{k}\left(x_{k}|\xi \right)}dx_{k}d\xi$$

$$\;=\sum_{i=1\;}^{p}\left(\iint f_{i}\left(x_{i}|\xi \right)g\left(\xi \right)\log\frac{f_{i}\left(x_{i}|\xi \right)}{f_{i}\left(x_{i}\right)}dx_{i}d\xi +\iint f_{i}\left(x_{i}\right)g\left(\xi \right)\log\frac{f_{i}\left(x_{i}\right)}{f_{i}\left(x_{i}|\xi \right)}dx_{i}d\xi \right)=\sum_{i=1}^{p}KL\left(X_{i},\xi \right)$$

 ☐

In model (1) with correlation matrix $\Phi =\left(\varphi_{ij}\right)$, we have

$$\;\mathrm{KL}\left(X_{i},\xi \;\right)=\frac{\sum_{k=1}^{m}\sum_{l=1}^{m}\lambda_{ik}\varphi_{kl}\lambda_{il}}{\sigma_{i}^{2}}$$

The above quantity is referred to as the contribution of ξ to $X_{i}$, and is denoted as $C\left(\xi \to X_{i}\right)$. Let $R_{i}$ be the multiple correlation coefficient of$\;X_{i\;}$and $\xi =\left(\xi_{j}\right)$. Then,

(11) $$\;C\left(\xi \to X_{i}\;\right)=\frac{R_{i}^{2}}{1-R_{i}^{2}}\left(=\mathrm{KL}\left(X_{i},\;\xi \right)\right)$$

From Theorem 1, we then have

(12) $$\;C\left(\xi \to X\;\right)=\sum_{i=1}^{p}\frac{R_{i}^{2}}{1-R_{i}^{2}}\left(=\mathrm{KL}\left(X,\;\xi \right)\right)$$

Hence, Theorem 1 gives the following decomposition of the contribution of ξ on X into those on the single manifest variables $X_{i}$ (11):

(13) $$\;C\left(\xi \to X\;\right)=\sum_{i=1}^{p}C\left(\xi \to X_{i}\right)$$

**Remark** **3.** Notice that in the denominator of (4), the total contribution of all factors $\xi_{i}$ is simply defined as the total sum assessed:

$$\;\sum_{l=1\;}^{m}C_{l}=\sum_{i=1}^{p}R_{i}^{2}$$

On the other hand, in the present approach, the total effect (contribution) of factor vector ξ on manifest variable vector X is decomposed into those of manifest variables $X_{i},\;\;\left(12\right)\;\mathrm{and}\;\left(13\right)$.

Let $X_{sub}$ be any sub-vector of manifest variable vector $X={\left(X_{1},X_{2},\ldots ,X_{p}\right)}^{T}$. Then, the contribution of factor vector ξ to $X_{sub}$ is defined by

$$\;C\left(\xi \to X_{sub\;}\right)=\mathrm{KL}\left(X_{sub},\xi \right)$$

From Theorem 1, we have the following corollary.

**Corollary** **1.** *Let* $X_{\left(1\right)}={\left(X_{i_{1}},X_{i_{2}},\ldots ,X_{i_{q}}\right)}^{T}$ *and* $X_{\left(2\right)}={\left(X_{j_{1}},X_{j_{2}},\ldots ,X_{j_{p-q}}\right)}^{T}$ *be a decomposition of manifest variable vector* $X={\left(X_{1},X_{2},\ldots ,X_{p}\right)}^{T}$ *, where q<p. Then, for factor analysis model (1), it follows that*

$$\;C\left(\xi \to X\right)=C\left(\xi \to X_{\left(1\right)}\right)+C\left(\xi \to X_{\left(2\right)}\right)\;\;C\left(\xi \to X_{(1)\;}\right)=\sum_{k=1}^{q}C\left(\xi \to X_{i_{k}}\right),\;\;C\left(\xi \to X_{\left(2\right)}\right)=\sum_{k=1}^{p-q}C\left(\xi \to X_{j_{k}}\right)$$

**Proof:**  From a similar discussion to the proof of Theorem 1, we have

$$\;\mathrm{KL}\left(X,\xi \right)=\mathrm{KL}\left(X_{\left(1\right)},\xi \right)+\mathrm{KL}\left(X_{\left(2\right)},\xi \right)\;\;\mathrm{KL}\left(X_{(1)\;},\xi \right)=\sum_{k=1}^{q}\mathrm{KL}\left(X_{i_{k}},\xi \right),\;\;\;\mathrm{KL}\left(X_{\left(2\right)},\xi \right)=\sum_{k=1}^{p-q}\mathrm{KL}\left(X_{j_{k}},\xi \right)$$

Hence, the corollary follows.

Next, the standardized total effects of single factors $\xi_{j\;}$on manifest variable vector X, that is, $e_{T}\left(\xi_{j}\to X\right)$, are calculated [8,10]. Let $\xi^{/j}={\left(\xi_{1},\xi_{2},\ldots ,\xi_{j-1},\xi_{j+1},\ldots ,\xi_{m}\right)}^{T}$; $f\left(x,\xi^{/j}|\xi_{j}\right)$ be the conditional density function of X and$\;\xi^{/j}$ given$\;\xi_{j}$; $f\left(x|\xi_{j}\right)\;$be the conditional density function of X given$\;\xi_{j}$; $\;g\left(\xi^{/j}|\xi_{j}\right)\;$be the conditional density function of$\;\xi^{/j}$given$\;\xi_{j}$; and $g_{j}\left(\xi_{j}\right)$ be the marginal density function of $\;\xi_{j}$. Then, we have

$$\begin{array}{l} \mathrm{KL}\left(X,\xi^{/j\;}|\xi_{j}\right)=\iint f\left(x,\xi \right)\log\frac{f\left(x,\xi^{/j}|\xi_{j}\right)}{f\left(x|\xi_{j}\right)g\left(\xi^{/j}|\xi_{j}\right)}dxd\xi^{/j}d\xi_{j}\\ \;+\iint f\left(x|\xi_{j}\right)g\left(\xi^{/j}|\xi_{j}\right)g_{j}\left(\xi_{j}\right)\log\frac{f\left(x|\xi_{j}\right)g\left(\xi^{/j}|\xi_{j}\right)}{f\left(x,\xi^{/j}|\xi_{j}\right)}dxd\xi^{/j}d\xi_{j}=\frac{\mathrm{tr}\tilde{\Omega }\Lambda \mathrm{cov}\left(\xi ,X|\xi_{j}\right)}{\left|\Omega \right|} \end{array}$$

where $\mathrm{cov}\left(\xi ,X|\xi_{j}\right)\;$is a m×p covariance matrix given $\xi_{j}$, of which the (k,i) elements are $\mathrm{cov}\left(\xi_{k},X_{i}|\xi_{j}\right)$. The standardized total effect$\;e_{T}\left(\xi_{j}\to X\right)$ is given by

$$\;e_{T}\left(\xi_{j}\to X\;\right)=\frac{\mathrm{KL}\left(X,\;\xi \right)-\mathrm{KL}\left(X,\xi^{/j}|\xi_{j}\right)}{\mathrm{KL}\left(X,\;\xi \right)+1}=\frac{\mathrm{tr}\tilde{\Omega }\Lambda \left(\mathrm{cov}\left(\xi ,X\right)-\mathrm{cov}\left(\xi ,X|\xi_{j}\right)\right)}{\;\mathrm{tr}\tilde{\Omega }\Lambda \mathrm{cov}\left(\xi ,X\right)+\left|\Omega \right|}$$

The standardized total effect$\;e_{T}\left(\xi_{j}\to X\right)$ [8] is interpreted as the contribution ratio of factor$\;\xi_{j}\;$in the whole entropy space of X, and in the present paper, it is denoted by $\tilde{RC}\left(\xi_{j}\to X\right)$. The contribution of factor$\;\xi_{j}$ measured in entropy is defined by

$$\;C\left(\xi_{j}\to X\;\right)=\;\mathrm{KL}\left(X,\;\xi \right)-\mathrm{KL}\left(X,\xi^{/j}|\xi_{j}\right)=\frac{\mathrm{tr}\tilde{\Omega }\Lambda \mathrm{cov}\left(\xi ,X\right)}{\left|\Omega \right|}-\frac{\mathrm{tr}\tilde{\Omega }\Lambda \mathrm{cov}\left(\xi ,X|\xi_{j}\right)}{\left|\Omega \right|}$$

As for (6), the relative contribution of factor$\;\xi_{j}$ on X is given by

$$\;\mathrm{RC}\left(\xi_{j}\to X\;\right)=\frac{\tilde{\mathrm{RC}}\left(\xi_{j}\to X\right)}{\tilde{\mathrm{RC}}\left(\xi \to X\right)}=\frac{C\left(\xi_{j}\to X\right)}{C\left(\xi \to X\right)}$$

Concerning factor contributions of $\xi_{j}$ on the single manifest variables $X_{i}$, that is, $C\left(\xi_{j}\to X_{i}\right)$, the following theorem can be stated.

**Theorem** **2.** *In the factor analysis model (1),*

$$\;C\left(\xi_{j}\to X\;\right)=\sum_{i=1}^{p}C\left(\xi_{j}\to X_{i}\right)$$

**Proof:**  From Theorem 1, it follows that

$$\;\mathrm{KL}\left(X,\xi^{/j\;}|\xi_{j}\right)=\sum_{i=1}^{p}\mathrm{KL}\left(X_{i},\xi^{/j}|\xi_{j}\right)$$

Then, we have

$$\;C\left(\xi_{j}\to X_{i}\;\right)=\mathrm{KL}\left(X_{i},\xi \right)-\mathrm{KL}\left(X_{i},\xi^{/j}|\xi_{j}\right)$$

and,

$$\begin{array}{l} C\left(\xi_{j}\to X\;\right)=\mathrm{KL}\left(X,\;\xi \right)-\mathrm{KL}\left(X,\xi^{/j}|\xi_{j}\right)\\ \;=\sum_{i=1}^{p}\mathrm{KL}\left(X_{i},\xi \right)-\sum_{i=1}^{p}\mathrm{KL}\left(X_{i},\xi^{/j}|\xi_{j}\right)\\ \;=\sum_{i=1}^{p}\left(KL\left(X_{i},\xi \right)-\mathrm{KL}\left(X_{i},\xi^{/j}|\xi_{j}\right)\right)=\sum_{i=1}^{p}C\left(\xi_{j}\to X_{i}\right) \end{array}$$

From the above theorem, we have the following corollary.

**Corollary** **2.** *Let*$X_{\left(1\right)}={\left(X_{i_{1}},X_{i_{2}},\ldots ,X_{i_{q}}\right)}^{T}$*and*$X_{\left(2\right)}={\left(X_{j_{1}},X_{j_{2}},\ldots ,X_{j_{p-q}}\right)}^{T}$*bedecomposition of manifest variable vector*$X={\left(X_{1},X_{2},\ldots ,X_{p}\right)}^{T}$*, where*q<p.

$$\;C\left(\xi_{j}\to X\;\right)=C\left(\xi_{j}\to X_{\left(1\right)}\right)+C\left(\xi_{j}\to X_{\left(2\right)}\right)\;\;C\left(\xi_{j}\to X_{(1)\;}\right)=\sum_{k=1}^{q}C\left(\xi_{j}\to X_{i_{k}}\right),\;\;C\left(\xi_{j}\to X_{\left(2\right)}\right)=\sum_{k=1}^{p-q}C\left(\xi_{j}\to X_{j_{k}}\right)$$

**Proof:**  From a similar discussion in the proof of Theorem 2, the corollary follows. ☐

**Remark** **4.** Let $X_{sub}$ be any sub-vector of manifest variable vector $X={\left(X_{1},X_{2},\ldots ,X_{p}\right)}^{T}$. By substituting X for $X_{sub}$ in the above discussion, $C\left(\xi \to X_{sub}\right)$, $C\left(\xi_{j}\to X_{sub}\right)$, $\tilde{\mathrm{RC}}\left(\xi_{j}\to X_{sub}\right)$, and $\mathrm{RC}\left(\xi_{j}\to X_{sub}\right)$ can be defined.

For orthogonal factor analysis models, the following theorem holds true.

**Theorem** **3.** *In factor analysis model (1), if common factors $\xi_{j}$ are statistically independent, then*

$$\;C\left(\xi \to X\;\right)=\sum_{j=1}^{m}\sum_{i=1}^{p}C\left(\xi_{j}\to X_{i}\right).$$

**Proof:**  From model (1), we have

$$\;C\left(\xi_{j}\to X_{i}\;\right)=\mathrm{KL}\left(X_{i},\xi \right)-\mathrm{KL}\left(X_{i},\xi^{/j}|\xi_{j}\right)=\frac{\lambda_{ij}^{2}}{\sigma_{i}^{2}}$$

This completes the theorem. ☐

From the above discussion, if common factors $\xi_{j}$ are statistically independent, (10) is derived. Moreover, we have

$$\;\tilde{\mathrm{RC}\;}\left(\xi_{j}\to X\right)=\frac{\mathrm{KL}\left(X,\xi \right)-\mathrm{KL}\left(X,\xi^{/j}|\xi_{j}\right)}{\mathrm{KL}\left(X,\xi \right)+1}=\frac{\sum_{i=1}^{p}\frac{\lambda_{ij}^{2}}{\sigma_{j}^{2}}}{\mathrm{KL}\left(X,\xi \right)+1}$$

This measure is the relative contribution ratio of $\xi_{j}\;$for the variation of X in entropy. The relative contributions of $\xi_{j}\;$ on X in entropy are calculated as follows:

$$\;\mathrm{RC}\left(\xi_{j}\to X\;\right)=\frac{C\left(\xi_{j}\to X\right)}{C\left(\xi \to X\right)}=\frac{\sum_{i=1}^{p}\frac{\lambda_{ij}^{2}}{\sigma_{i}^{2}}}{\sum_{j=1}^{m}\sum_{i=1}^{p}\frac{\lambda_{ij}^{2}}{\sigma_{i}^{2}}}$$

**Remark** **5.** It is difficult to use OCD for assessing factor contributions, because |Σ| cannot be decomposed as in the above discussion.

## 5. Numerical Example

In order to illustrate the present method, we use the data shown in Table 1 [12]. In this table, manifest variables $X_{1},\;X_{2},\;\mathrm{and}\;X_{3}$ are subjects in liberal arts and variables $X_{4}\;\mathrm{and}\;X_{5}$ are those in sciences. First, orthogonal factor analysis (varimax method by S-PLUS ver. 8.2) is applied to the data and the results are illustrated in Table 2. From the estimated factor loadings, the first factor is interpreted as an ability relating to liberal arts, and the second factor as that for sciences. According to the factor contributions $C\left(\xi_{j}\to X\right)$ shown in Table 3, the contribution of factor $\xi_{2}$ is about twice as big than that of factor $\xi_{1}$ from a view point of entropy, and from the relative contributions $\tilde{\mathrm{RC}}\left(\xi_{j}\to X\right)$, about 30% of variation of manifest variable vector X in entropy is explained by factor $\xi_{1}$ and about 60% by factor $\xi_{2}$. The relative contribution $\tilde{\mathrm{RC}}\left(\xi \to X\right)$ in Table 3 implies about 90% of the entropy of manifest variable vector X is explained by the two factors. On the other hand, in the conventional method, the measured factor contributions of $\xi_{1}$ and $\xi_{2}$, that is, $C_{j}$, are almost equal (Table 4). As discussed in the present paper, the conventional method is intuitive and does not have any logical foundation for multidimensionally measuring contributions of factors to manifest variable vectors. Table 5 decomposes “the contribution of ξ to X” into components $C\left(\xi_{j}\to X_{i}\right)$. The contribution of $\xi_{2}$ to $X_{5}$ is prominent compared with the other contributions.

From the discussion in the previous section, the contributions of factors are flexibly calculated. For example, it is reasonable to divide the manifest variable vector into $X_{\left(1\right)}=\left(X_{1},X_{2},X_{3}\right)$ and $X_{\left(2\right)}=\left(X_{4},X_{5}\right)$, because the first sub-vector is related to the liberal arts and the second one to the sciences. First, the contributions of $\xi_{1}$ and $\xi_{2}$ to $X_{\left(1\right)}$ are calculated according to the present method, and the details are given as follows:

$$\;C\left(\xi_{1}\to X_{\left(1\right)}\right)=C\left(\xi_{1}\to X_{1}\right)+C\left(\xi_{1}\to X_{2}\right)+C\left(\xi_{1}\to X_{3}\right)=0.72+1.49+0.72=2.93\;\;\;C\left(\xi_{2}\to X_{(1)\;}\right)=0.30+0.15+0.00=0.45$$

(14) $$\;C\left(\xi \to X_{\left(1\right)}\right)=C\left(\xi_{1}\to X_{\left(1\right)}\right)+\;C\left(\xi_{2}\to X_{\left(1\right)}\right)=2.93+0.45=3.38\;\;\tilde{\mathrm{RC}\;}\left(\xi \to X_{\left(1\right)}\right)=\frac{C\left(\xi \to X_{\left(1\right)}\right)}{C\left(\xi \to X_{\left(1\right)}\right)+1}=\frac{3.38}{3.38+1}=0.77$$

(15) $$\;\tilde{\mathrm{RC}\;}\left(\xi_{1}\to X_{\left(1\right)}\right)=\frac{C\left(\xi_{1}\to X_{\left(1\right)}\right)}{C\left(\xi \to X_{\left(1\right)}\right)+1}=\frac{2.93}{3.38+1}=0.67$$

(16) $$\;\tilde{\mathrm{RC}\;}\left(\xi_{2}\to X_{\left(1\right)}\right)=\frac{0.45}{3.38+1}=0.10$$

(17) $$\;\mathrm{RC}\left(\xi_{1}\to X_{(1)\;}\right)=\frac{C\left(\xi_{1}\to X_{\left(1\right)}\right)}{C\left(\xi \to X_{\left(1\right)}\right)}=\frac{2.93}{2.93+0.45}=0.87$$

(18) $$\;\mathrm{RC}\left(\xi_{2}\to X_{(1)\;}\right)=\frac{0.45}{2.93+0.45}=0.13$$

From (14), 77% of the entropy of manifest variable sub-vector $X_{\left(1\right)}$ are explained by the two factors, in which 67% of that are explained by factor $\xi_{1}$ (15) and 10% by factor $\xi_{2}\;\left(16\right)$. From the relative contributions (17) and (18), 87% of the total contribution of the two factors are made by factor $\xi_{1}$ and 13% by factor $\xi_{2}$.

On the other hand, the contributions of $\xi_{1}$ and $\xi_{2}$ on $X_{\left(2\right)}=\left(X_{4},X_{5}\right)$ are calculated as follows:

$$\;C\left(\xi_{1}\to X_{\left(2\right)}\right)=C\left(\xi_{2}\to X_{4}\right)+C\left(\xi_{2}\to X_{5}\right)=0.19+0.00=0.19\;\;\;C\left(\xi_{2}\to X_{(2)\;}\right)=0.63+5.14=5.77$$

(19) $$\;C\left(\xi \to X_{\left(2\right)}\right)=C\left(\xi_{1}\to X_{\left(2\right)}\right)+\;C\left(\xi_{2}\to X_{\left(2\right)}\right)=0.19+5.77=5.96\;\;\tilde{\mathrm{RC}\;}\left(\xi \to X_{\left(2\right)}\right)=\frac{C\left(\xi \to X_{\left(2\right)}\right)}{C\left(\xi \to X_{\left(2\right)}\right)+1}=\frac{5.96}{5.96+1}=0.86$$

(20) $$\;\tilde{\mathrm{RC}\;}\left(\xi_{1}\to X_{\left(2\right)}\right)=\frac{C\left(\xi_{1}\to X_{\left(2\right)}\right)}{C\left(\xi \to X_{\left(2\right)}\right)+1}=\frac{0.19}{5.96+1}=0.03$$

(21) $$\;\tilde{\mathrm{RC}\;}\left(\xi_{2}\to X_{\left(2\right)}\right)=\frac{5.77}{5.96+1}=0.83$$

(22) $$\;\mathrm{RC}\left(\xi_{1}\to X_{(2)\;}\right)=\frac{C\left(\xi_{1}\to X_{\left(2\right)}\right)}{C\left(\xi \to X_{\left(2\right)}\right)}=\frac{0.19}{5.96}=0.03$$

(23) $$\;\mathrm{RC}\left(\xi_{2}\to X_{(2)\;}\right)=\frac{5.77}{5.96}=0.97$$

From (19), 86% of entropy of manifest variable sub-vector $X_{\left(2\right)}$ is explained by the two factors, in which 3% of the entropy are explained by factor $\xi_{1}$ (20) and 83% by factor $\xi_{2}$ (21). The contribution ratios of the factors to sub-vector $X_{\left(2\right)}$ are calculated in (22) and (23). Ninety-seven percent of the entropy was made by factor $\xi_{2}$.

Second, factor contributions in an oblique case are calculated. The estimated factor loadings and the correlation matrix of factors based on the covarimin method are shown in Table 6 and Table 7, respectively. Based on factor loadings in Table 6, factor $\xi_{1}$ is interpreted as an ability for subjects in the liberal arts and factor $\xi_{2}$ as an ability for subjects in sciences. The results are similar to those in the orthogonal case mentioned above, because the correlation between the factors is not strong. Table 8 shows the decomposition of C(ξ→X) based on Theorems 1 and 2. In this case, it is noted that $C\left(\xi \to X\right)\neq C\left(\xi_{1}\to X\right)+C\left(\xi_{2}\to X\right)$; however, $C\left(\xi \to X\right)=\sum_{i=1}^{5}C\left(\xi \to X_{i}\right)$. According to the table, the contributions of $\xi_{1}$ and $\xi_{2}$ to sub-vectors of manifest variable vector X can also be calculated as in the above orthogonal factor analysis. Table 9 illustrates the contributions of factors on manifest variable vector X. Factor $\xi_{1}$ explains 42% of the entropy of X and factor $\xi_{2}$ explains 71%.

## 6. Discussion

For orthogonal factor analysis models, the conventional method measures factor contributions (effects) by the sums (totals) of squared factor loadings related to the factors (2); however, there is no logical foundation for how they can be interpreted. It is reasonable to measure factor contributions as the effects of factors on the manifest variable vector concerned. The present paper has proposed a method of measuring factor contributions through entropy, that is, applying an entropy-based path analysis approach. The method measures the contribution of factor vector ξ to manifest variable vector X and decomposes it into those of factors $\xi_{j}$ to manifest variables $X_{i}$ and/or those to sub-vectors of X. Comparing (2) and (10), for standardization of unique factor variances $\sigma_{i}^{2}=1$, the present method equals to the conventional method. As discussed in this paper, the present method can be employed in oblique factor analysis as well, and it has been illustrated in a numerical example. The present method has a theoretical basis for measuring factor contributions in a framework of entropy, and it is a novel approach for factor analysis. The present paper confines itself to the usual factor analysis model. A more complicated model with a mixture of normal factor analysis models [13] is excluded, and a further study is needed to apply the entropy-based method to the model.

## Figures and Tables

**Table 1:** Data for illustrating factor analysis.

Subject	$\mathrm{Japanese}\;X_{1}\;$	$\mathrm{English}\;X_{2}\;$	$\mathrm{Social}\;X_{3}\;$	$\mathrm{Mathematics}\;X_{4}\;$	$\mathrm{Science}\;X_{5}\;$
1	64	65	83	69	70
2	54	56	53	40	32
3	80	68	75	74	84
4	71	65	40	41	68
5	63	61	60	56	80
6	47	62	33	57	87
7	42	53	50	38	23
8	54	17	46	58	58
9	57	48	59	26	17
10	54	72	58	55	30
11	67	82	52	50	44
12	71	82	54	67	28
13	53	67	74	75	53
14	90	96	63	87	100
15	71	69	74	76	42
16	61	100	92	53	58
17	61	69	48	63	71
18	87	84	64	65	53
19	77	75	78	37	44
20	57	27	41	54	30

**Table 2:** Factor loadings of orthogonal factor analysis ($\chi^{2}=0.55,\;df=1,\;P=0.45).$

	$X_{1}$	$X_{2}$	$X_{3}$	$X_{4}$	$X_{5}$
$\xi_{1}$	0.60	0.75	0.65	0.32	0.00
$\xi_{2}$	0.39	0.24	0.00	0.59	0.92
uniqueness	0.50	0.38	0.58	0.55	0.16

**Table 3:** Factor contributions based on entropy (orthogonal case).

	$\;\xi_{1}\;$	$\;\xi_{2}\;$	Total
$C\left(\xi_{j}\to X\right)$	3.11	6.23	9.34=C(ξ→X)
$\tilde{\mathrm{RC}}\left(\xi_{j}\to X\right)$	0.30	0.60	$0.90=\tilde{RC}\left(\xi \to X\right)$
$\mathrm{RC}\left(\xi_{j}\to X\right)$	0.33	0.67	1

**Table 4:** Factor contributions with the conventional method.

	$\;\xi_{1}\;$	$\;\xi_{2}\;$	Total
$C_{j}$	1.44	1.39	2.83
${\tilde{\mathrm{RC}}}_{j}$	0.29	0.28	0.57
${\mathrm{RC}}_{j}$	0.51	0.49	1

**Table 5:** Decomposition of factor contribution C(ξ→X) into $C\left(\xi_{j}\to X_{i}\right).$

	$\;X_{1}\;$	$\;X_{2}\;$	$\;X_{3}\;$	$\;X_{4}\;$	$\;X_{5}\;$	$\mathrm{Total}\;=C\left(\xi_{j}\to X\;\right)$
$\xi_{1}$	0.72	1.49	0.72	0.19	0.00	3.11
$\xi_{2}$	0.30	0.15	0	0.63	5.14	6.23
total$\;=C\left(\xi \to X_{i}\right)$	1.01	1.64	0.72	0.82	5.14	9.34

**Table 6:** Factor loadings of oblique factor analysis ($\chi^{2}=0.55,\;df=1,\;P=0.45).$

	$\;X_{1}\;$	$\;X_{2}\;$	$\;X_{3}\;$	$\;X_{4}\;$	$\;X_{5}\;$
$\xi_{1}$	0.59	0.77	0.68	0.29	0
$\xi_{2}$	0.24	0.00	−0.12	0.52	0.92
uniqueness	0.50	0.41	0.58	0.55	0.16

**Table 7:** Correlation matrix of factors.

	$\;\xi_{1}\;$	$\;\xi_{2}\;$
$\xi_{1}$	1	0.315
$\xi_{2}$	0.315	1

**Table 8:** Decomposition of factor contribution C(ξ→X) into $C\left(\xi_{j}\to X_{i}\right)$ (oblique case).

	$\;X_{1}\;$	$\;X_{2}\;$	$\;X_{3}\;$	$\;X_{4}\;$	$\;X_{5}\;$	$\;\mathrm{Total}=C\left(\xi_{j}\to X\;\right)$
$\xi_{1}$	0.90	1.44	0.70	0.37	0.54	3.95
$\xi_{2}$	0.37	0.14	0.01	0.68	5.43	6.65
$C\left(\xi \to X_{i}\right)$	1.01	1.44	0.73	0.82	5.43	C(ξ→X)=9.43

**Table 9:** Factor contributions based on entropy (oblique case).

	$\;\xi_{1}\;$	$\;\xi_{2}\;$	Effect of ξ on X
$C\left(\xi_{j}\to X\right)$	3.95	6.65	C(ξ→X)=9.43
$\tilde{\mathrm{RC}}\left(\xi_{j}\to X\right)$	0.38	0.64	$\tilde{\mathrm{RC}}\left(\xi \to X\right)=0.90$
$\mathrm{RC}\left(\xi_{j}\to X\right)$	0.42	0.71	
